# Synaptic localisation of SRF coactivators, MKL1 and MKL2, and their role in dendritic spine morphology

**DOI:** 10.1038/s41598-017-18905-7

**Published:** 2018-01-15

**Authors:** Marisa Kaneda, Hiroyuki Sakagami, Yamato Hida, Toshihisa Ohtsuka, Natsumi Satou, Yuta Ishibashi, Mamoru Fukuchi, Anna Krysiak, Mitsuru Ishikawa, Daisuke Ihara, Katarzyna Kalita, Akiko Tabuchi

**Affiliations:** 1 Laboratory of Molecular Neurobiology, Graduate School of Medicine and Pharmaceutical Sciences, University of Toyama, 2630 Sugitani, Toyama, 930–0194 Japan; 2 Department of Anatomy, Kitasato University School of Medicine, Sagamihara, Kanagawa, 252–0734 Japan; 3 Department of Biochemistry, Graduate School of Medicine, University of Yamanashi, 1110 Shimokato, Chuo Yamanashi, 409–3898 Japan; 4 Present Address: Laboratory of Molecular Neuroscience, Faculty of Pharmacy, Takasaki University of Health and Welfare, 60 Nakaorui-machi, Takasaki Gunma, 370–0033 Japan; 5 Laboratory of Neurobiology, Department of Molecular and Cellular Neurobiology, Nencki Institute of Experimental Biology, Polish Academy of Sciences, 3 Pasteur Street, 02–093 Warsaw, Poland; 6 Present Address: Department of Physiology, Keio University, School of Medicine, 35 Shinanomachi, Shinjuku-ku Tokyo, 160–8582 Japan

## Abstract

The megakaryoblastic leukaemia (MKL) family are serum response factor (SRF) coactivators, which are highly expressed in the brain. Accordingly, MKL plays important roles in dendritic morphology, neuronal migration, and brain development. Further, nucleotide substitutions in the *MKL*1 and *MKL*2 genes are found in patients with schizophrenia and autism spectrum disorder, respectively. Thus, studies on the precise synaptic localisation and function of MKL in neurons are warranted. In this study, we generated and tested new antibodies that specifically recognise endogenously expressed MKL1 and MKL2 proteins in neurons. Using these reagents, we biochemically and immunocytochemically show that MKL1 and MKL2 are localised at synapses. Furthermore, shRNA experiments revealed that postsynaptic deletion of *MKL1* or *MKL*2 reduced the percentage of mushroom- or stubby-type spines in cultured neurons. Taken together, our findings suggest that MKL1 and MKL2 are present at synapses and involved in dendritic spine maturation. This study may, at least in part, contribute to better understanding of the molecular mechanisms underlying MKL-mediated synaptic plasticity and neurological disorders.

## Introduction

Serum response factor (SRF) is a transcription factor that regulates a series of immediate early genes (such as *c*-*fos* and *egr*-*1*), cytoskeletal genes (such as β-actin), and the neuronal immediate early and cytoskeletal gene, activity-regulated cytoskeleton-associated protein (*Arc*)^1,2^ by binding to a CArG [CC(A/T)_6_GG] box within the promoter region of target genes^3^. Conventional and conditional knock-out of SRF *in vivo* revealed that SRF is involved in a vast array of physiological or pathological processes^4^. Accordingly, functional significance of SRF in the brain has been reported. For example, SRF contributes to neuronal activity-dependent gene expression, long-term potentiation (LTP)^1^, long-term depression (LTD)^5^, formation of proper neuronal circuits^6,7^, and regulation of epilepsy^8^.

Serum response factor exerts a regulatory effect on gene transcription in concert with cofactors. Ternary complex factor (TCF) is a representative SRF cofactor, which binds to a specific sequence adjacent to the SRF binding site^9^. Other SRF cofactors include the myocardin and megakaryoblastic leukaemia (MKL) family. The MKL family consists of MKL1/megakaryocytic acute leukaemia (MAL)/myocardin-related transcription factor-A (MRTF-A)/basic SAP and coiled-coil domain (BSAC), and MKL2/MRTF-B^10–15^. MKL has actin-binding RPEL motifs and a transcriptional activation domain^16^. Although MKL1 binds to G-actin in the cell resting state, MKL translocates into the nucleus to bind and activate SRF after activation of Rho small GTPases and actin polymerisation^16^. MKL is highly expressed in the brain^17–19^ and double knock-out of *MKL*1/*MKL*2 in the brain displays phenotypical similarity to SRF conditional knock-out mice^20^. These studies suggest that in addition to SRF, its cofactors (specifically MKL family members), play important brain roles. Recent observations suggest that mutations or single nucleotide polymorphisms (SNPs) of *MKL1* and *MKL*2 genes are found in patients with schizophrenia^21,22^ and autism spectrum disorder (ASD)^23,24^, respectively. Therefore, elucidating the molecular mechanism by which MKL family members control neuronal function may shed light on understanding brain function and disorders^25^. Although MKL regulates dendritic morphology at the cellular level^17,18,26^, subcellular localisation of MKL1 and MKL2, and their role in spine maturation remain unknown. In this study, we generated anti-MKL1- and MKL2-specific antibodies that are suitable for western blotting and immunostaining. Using these newly characterised antibodies, we show synaptic localisation of MKL1 and MKL2 proteins in cultured neurons and a synaptosomal fraction. Furthermore, we show that MKL1 and MKL2 control dendritic spine morphology by RNA interference-mediated knock-down in cultured neurons. Neuronal knock-down of MKL1 and MKL2 reduced the percentage of mushroom- or stubby-shaped spines, suggesting novel roles of MKL in spine maturation.

## Results

### Evaluation of new antibodies by western blotting and immunostaining

**Figure 1:**
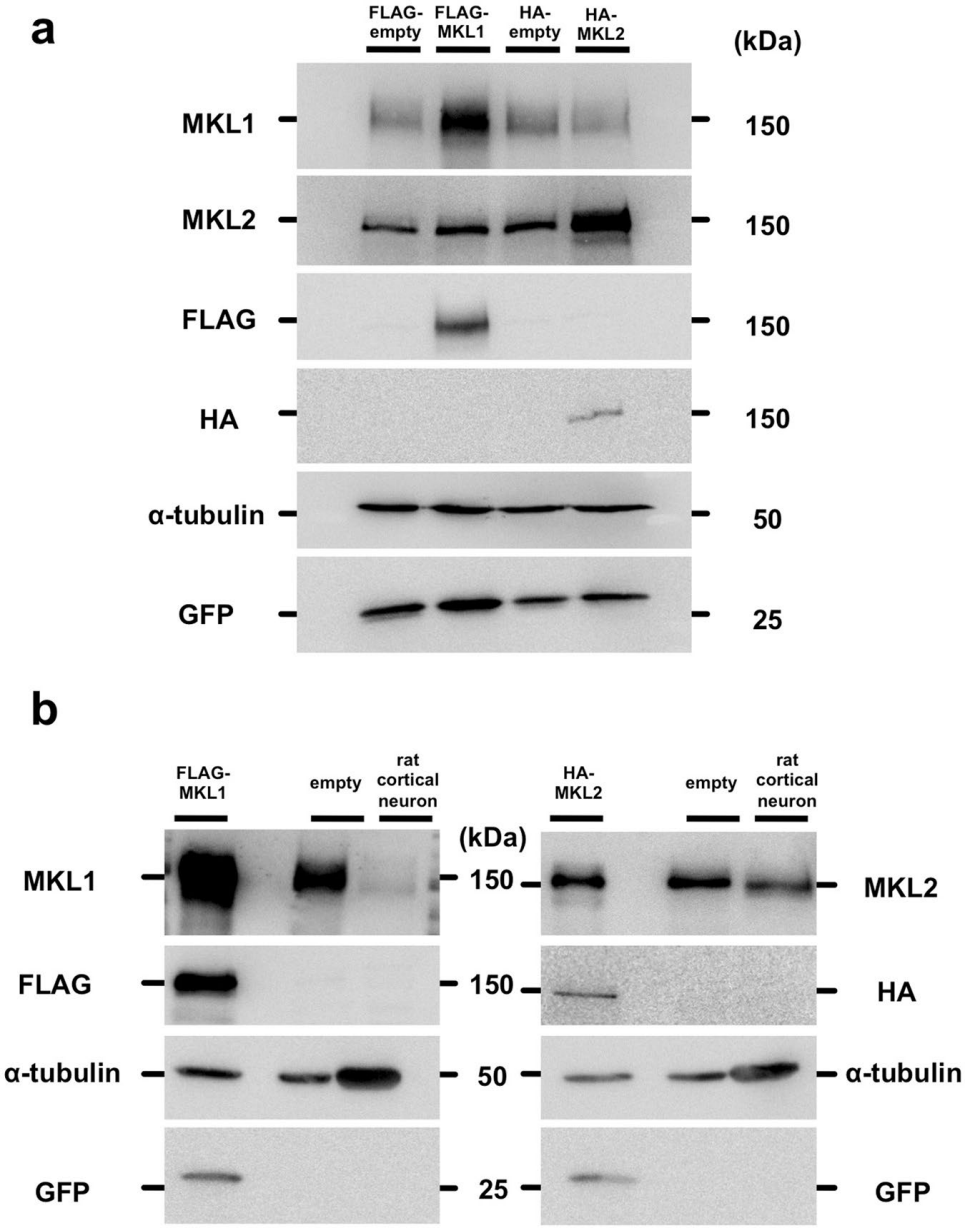
Specific detection of MKL1 and MKL2 by anti-MKL1 and anti-MKL2 antibodies. (**a**) pEGFP-C1 vector and pFLAG-CMV2 vector (FLAG-empty) or pFLAG-CMV2-MKL1 vector (FLAG-MKL1), pCMV-HA vector (HA-empty) or pCMV-HA-MKL2 vector (HA-MKL2) were co-transfected into NIH3T3 cells. Cell lysates were lysed 24 hours after transfection. Anti-MKL1 (MKL1) and anti-MKL2 (MKL2) antibody were used for detection of exogenous and endogenous MKL1 and MKL2, respectively. Anti-FLAG and anti-HA antibodies were used for detection of exogenous MKL1 and MKL2, respectively. Anti-α-tubulin and anti-GFP antibodies were used as internal (loading) controls. (**b**) Detection of endogenous MKL1 and MKL2 in cortical neurons. Left-most lanes: cell lysates from NIH3T3 cells overexpressing FLAG-MKL1 and HA-MKL2. Lane 2: cell lysates from untransfected NIH3T3 cells. Lane 3: cell lysates from rat cortical neurons at 7 days in culture. Full-length blots are presented in Supplementary Figure S5.

A previous study demonstrated that the N-terminal region of MKL1 shows high homology to MKL2^13^. Therefore, to obtain MKL1 or MKL2 antibodies that specifically recognise MKL1 and MKL2, we chose the C-terminal regions of MKL1 and MKL2 as antigens (which show low homology [37.2%] to each other), and include transcriptional activation domains. First, we evaluated specificity of these new MKL1 and MKL2 antibodies. Cell lysates were prepared from NIH3T3 cells expressing FLAG-empty vector (FLAG-empty), FLAG-MKL1, HA-empty vector (HA-empty), or HA-MKL2, and subjected to western blotting (Fig. 1a). MKL1 antibody strongly reacted with samples expressing FLAG-MKL1 (Fig. 1a, lane 2) and appeared to detect endogenous MKL1 (Fig. 1a, lanes 1, 3, and 4). Whereas, MKL2 antibody strongly reacted with the sample expressing HA-MKL2 (Fig. 1a, lane 4) and appeared to recognise endogenous MKL2 (Fig. 1a, lanes 1, 2, and 3). Even though HA-MKL2 was expressed in the sample (Fig. 1a, lane 4), the intensity of the band detected by MKL1 was the same as the empty vector bands (Fig. 1a, lanes 1 and 3). Similarly, even though FLAG-MKL1 was expressed in the sample (Fig. 1a, lane 2), the intensity of the band detected by MKL2 was the same as the empty vector bands (Fig. 1a, lanes 1 and 3). These results suggest that MKL1 antibody does not cross-react with MKL2, and MKL2 antibody does not cross-react with MKL1. We also determined if these antibodies recognise rat MKL1 and MKL2 (Fig. 1b). MKL1 and MKL2 antibodies detected cell lysate samples from 7-day cultured cortical neurons (left panel, lane 4 and right panel, lane 4, respectively). In addition to western blotting, we demonstrated that antibodies generated against MKL1 and MKL2 are suitable for immunostaining of endogenous MKL1 and MKL2 (Supplementary Figs S1–S2).

### Nuclear translocation of MKL in NIH3T3 cells and cortical neurons

**Figure 2:**
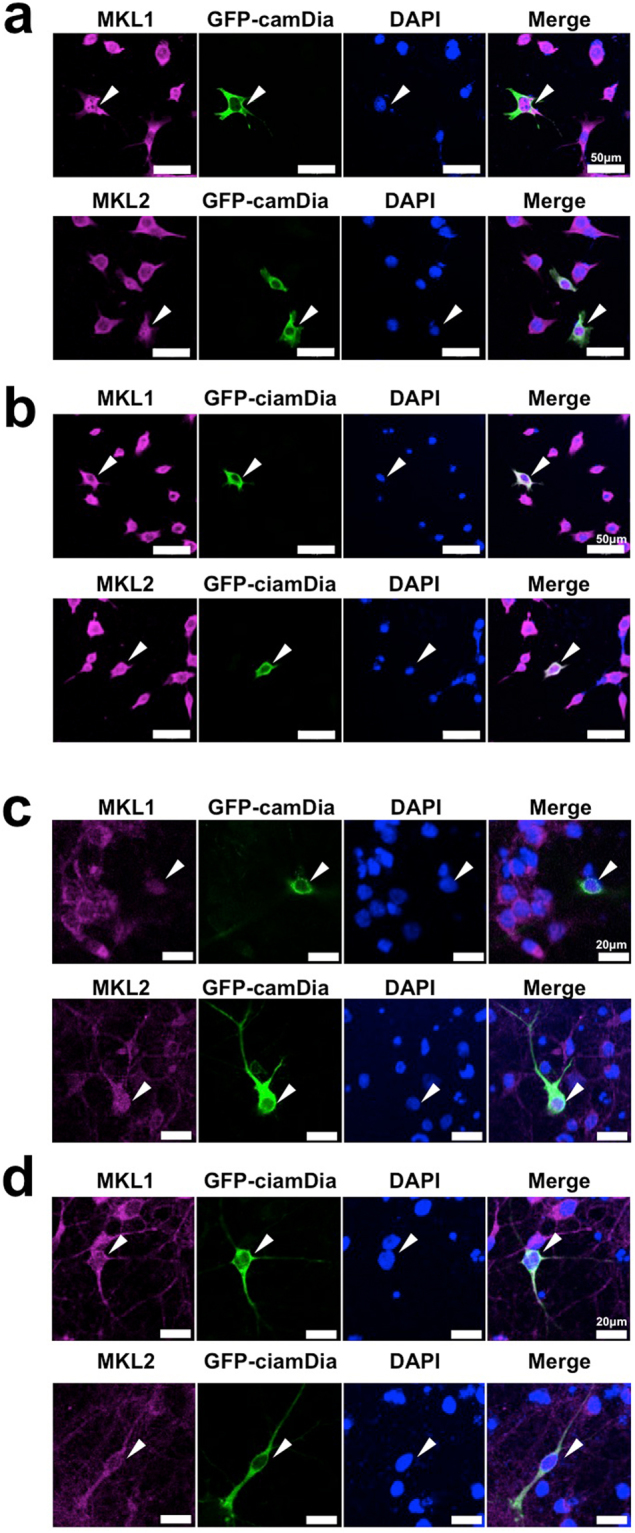
Nucleocytoplasmic staining of MKL in NIH3T3 cells and cortical neurons expressing a Rho effector, mDia. NIH3T3 cells were transfected with GFP-tagged constitutively active (ca) mDia (**a**) or constitutively inactive (cia) mDia (**b**). Twenty-four hours later, cells were immunostained with anti-GFP and anti-MKL1 or anti-MKL2 antibodies. Cortical neurons (7 days in culture) were transfected with camDia (4 μg/well, **c**) or ciamDia (4 μg/well, **d**). Twenty-four hours later, cells were immunostained with anti-GFP and anti-MKL1 or anti-MKL2 antibodies. Arrowheads indicate GFP-mDia-positive cells. Data regarding the cellular localisation of MKL are presented in Supplementary Figure S3.

Rho signalling promotes nuclear translocation of MKL1^16,27^. These findings encouraged us to detect nuclear translocation of MKL1 and MKL2 using our antibodies. Thus, we examined whether nuclear translocation of MKL1 and MKL2 was induced by constitutively active (ca) mDia, which is a downstream effector of Rho (Fig. 2, Supplementary Fig. S3). In NIH3T3 cells expressing camDia, the cellular distribution of MKL1 was 56%, 11% and 33% in the nucleus, cytoplasm, or both, respectively (Supplementary Fig. S3a). Conversely, in NIH3T3 cells expressing constitutively inactive (cia)mDia, a negative control, the distribution of MKL1 was 5%, 65%, and 30% in the nucleus, cytoplasm, or both, respectively (Supplementary Fig. S3b). The distribution of MKL2 in NIH3T3 cells expressing camDia was 50%, 12%, and 38% in the nucleus, cytoplasm, or both, respectively (Supplementary Fig. S3c). In NIH3T3 cells expressing ciamDia, 24%, 30%, and 46% of MKL2 was distributed in the nucleus, cytoplasm, or both, respectively (Supplementary Fig. S3d). Therefore, camDia effectively induced nuclear translocation of MKL1 and MKL2 in NIH3T3 cells. In cortical neurons expressing camDia, the distribution of MKL1 was 2%, 64%, and 34% in the nucleus, cytoplasm, or both, respectively (Supplementary Fig. S3e). In cortical neurons expressing ciamDia 1%, 88%, and 11% of MKL1 was distributed in the nucleus, cytoplasm, or both, respectively (Supplementary Fig. S3f). The distribution of MKL2 in cortical neurons expressing camDia was 8%, 43%, and 49% in the nucleus, cytoplasm, or both, respectively (Supplementary Fig. S3g). In cortical neurons expressing ciamDia, 7%, 61%, 32% of MKL2 was found in the nucleus, cytoplasm, or both, respectively (Supplementary Fig. S3h). Thus, the percentages of MKL1 and MKL2, localised in the nucleus only, were very small, and did not alter much even though camDia was expressed in cortical neurons. However, the levels of MKL1 and MKL2 in both the nucleus and cytoplasm were increased, and in turn, the levels in the cytoplasm were decreased in the presence of camDia. These findings indicate that the MKL1 or MKL2 antibodies are suitable for nucleocytoplasmic staining in NIH3T3 cells and cortical neurons.

### Biochemical detection of MKL1 and MKL2 in subcellular fractions of rat brain

**Figure 3:**
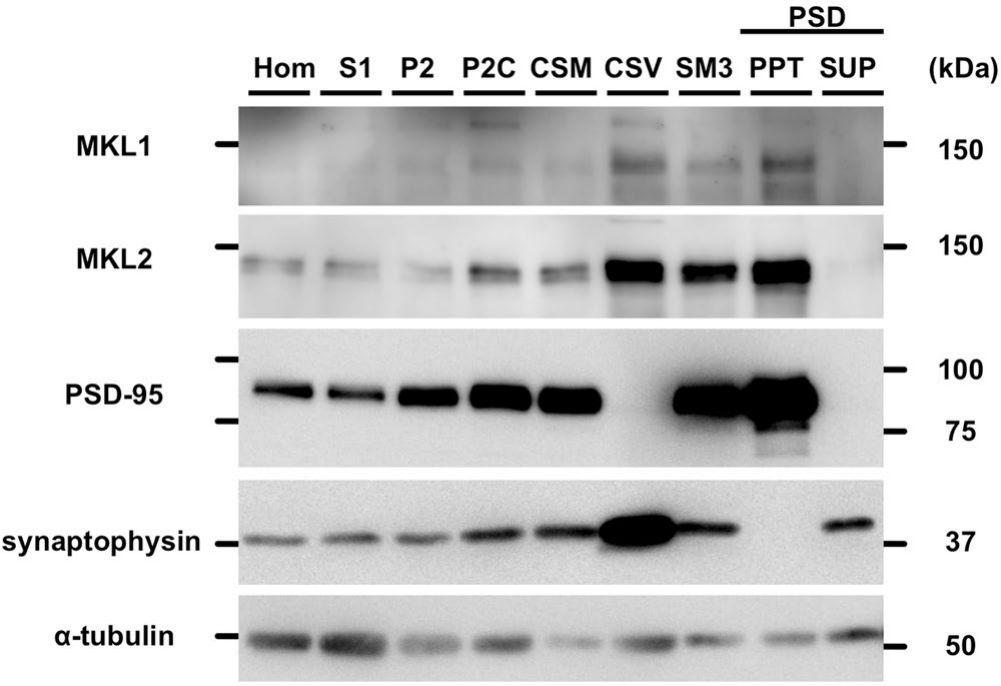
Detection of endogenous MKL1 and MKL2 protein in subcellular fractions of rat brains. Subcellular fractionation was performed from homogenates of 6-week-old male rat brain. Each protein (5 μg) was analysed by western blotting using anti-MKL1, anti-MKL2, anti-PSD-95 (postsynaptic marker), anti-synaptophysin (presynaptic marker), and anti-α-tubulin antibodies. Hom: homogenate; S1: crude synaptosomal fraction; P2: crude membrane fraction; P2C: synaptosomal fraction; CSM: crude synaptic membrane fraction; CSV: crude synaptic vesicle fraction; SM3: synaptic membrane fraction 3; PSD: postsynaptic density fraction; PPT: 1% Triton X-100-insoluble fraction of PSD; SUP: 1% Triton X-100-soluble fraction of PSD. Full-length blots are presented in Supplementary Figure S6.

Next, we investigated subcellular localisation of MKL1 and MKL2 protein in brain extracts and cortical neurons. Initially, we performed biochemical detection of MKL1 and MKL2 protein by subcellular fractionation of rat brain. A strong signal was obtained from precipitates of postsynaptic density (PSD) fractions (PPT) using anti-PSD-95 antibody, a postsynaptic marker (Fig. 3, PSD-95). In addition, a strong signal was obtained from the crude synaptic vesicle (CSV) fraction using anti-synaptophysin antibody, a presynaptic marker (Fig. 3, synaptophysin). These results indicate successful subcellular fractionation. Both MKL1 and MKL2 were enriched in PSD (PPT), synaptic membrane 3 (SM3), and CSV fractions (Fig. 3, MKL1 and MKL2), indicating pre-and postsynaptic localisation of MKL1 and MKL2 proteins.

### Synaptic localisation of a portion MKL1 and MKL2 by immunocytochemistry

**Figure 4:**
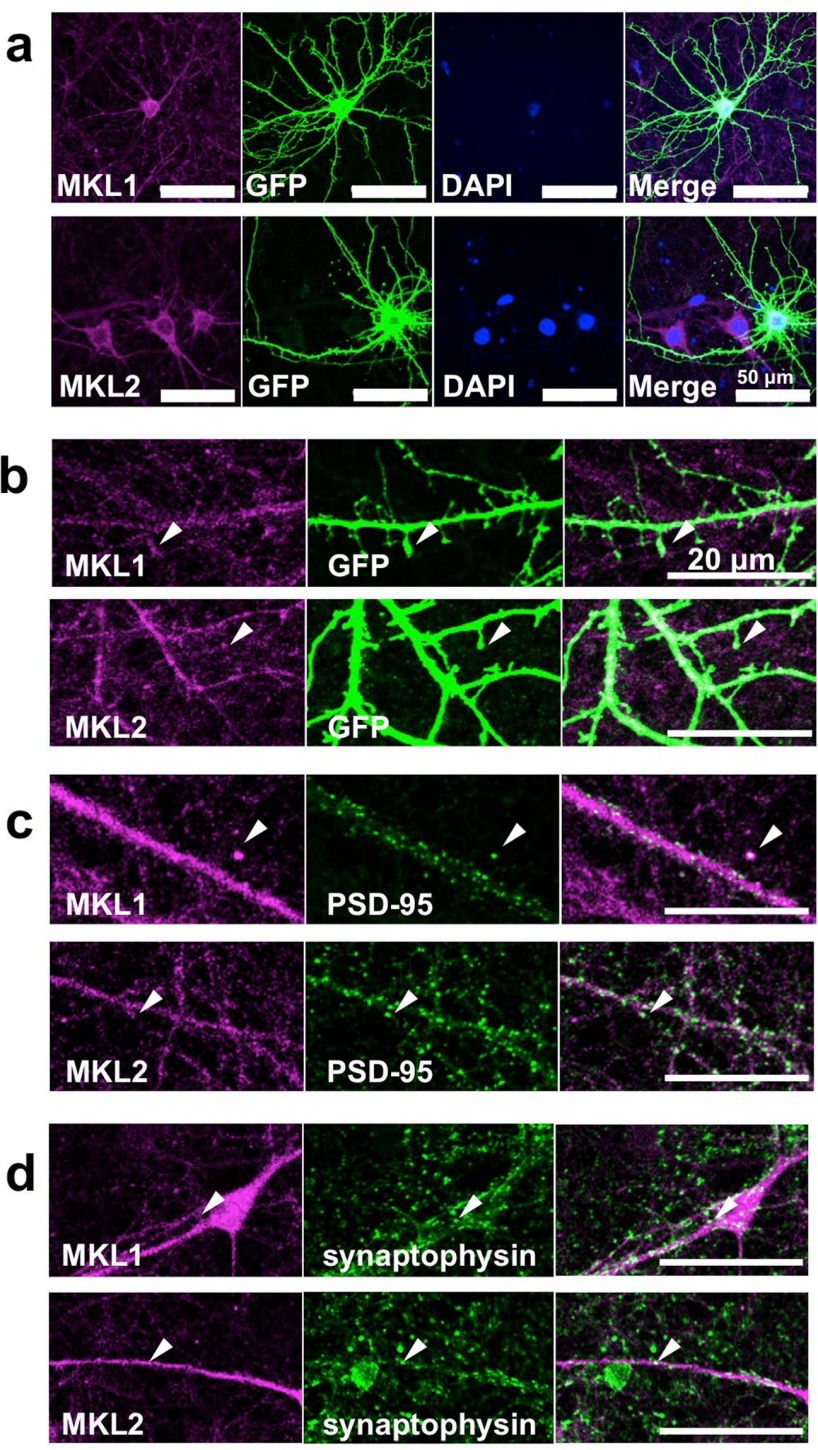
Immunocytochemical detection of MKL1 and MKL2 protein in cortical neurons with synapses. (**a**) Immunostaining of mature, cortical neuronal cultures. pEGFP-C1 vector (4 μg/well) was transfected into cortical neurons at 18 days in culture. Three days later, cells were subjected to immunostaining using anti-GFP and anti-MKL1 or anti-MKL2 antibodies. (**b**) High magnification of GFP-positive cortical neurons at 21 days in culture. Arrowheads indicate dendritic spines. (**c**,**d**) Synaptic staining of MKL1 and MKL2 in mature, cortical neurons. Cortical neurons at 21 days in culture were immunostained using anti-MKL1, anti-MKL2, anti-postsynaptic density (PSD)-95, and anti-synaptophysin antibodies. MKL and PSD-95 signals merged. MKL and synaptophysin signals also merged.

Subsequently, we performed immunocytochemical detection of MKL1 and MKL2 protein in mature cortical neurons. Neuronal morphology was visualised by expressing green fluorescent protein (GFP) followed by immunostaining with GFP antibody and MKL1 or MKL2 antibody (Fig. 4a). We found MKL1 and MKL2 protein enriched in cortical neuronal cell bodies, with punctate staining for both MKL1 and MKL2 protein observed in dendrites. Puncta appeared to be localised within nuclei. Higher magnified images of dendrites revealed that some puncta were localised at GFP-positive dendritic spines (Fig. 4b, arrowheads) where PSD-95 and MKL1 or MKL2 signals merged (Fig. 4c, arrowheads), supporting postsynaptic localisation of MKL1 or MKL2. Furthermore, synaptophysin and MKL1 or MKL2 signals also merged (Fig. 4d, arrowheads), demonstrating pre-synaptic localisation of MKL1 or MKL2 protein as well. However, not all puncta stained with anti-MKL1 or anti-MKL2 antibodies colocalised with puncta stained with anti-PSD-95 or anti-synaptophysin antibodies.

### Functional role of MKL1 and MKL2 in dendritic spine maturation

**Figure 5:**
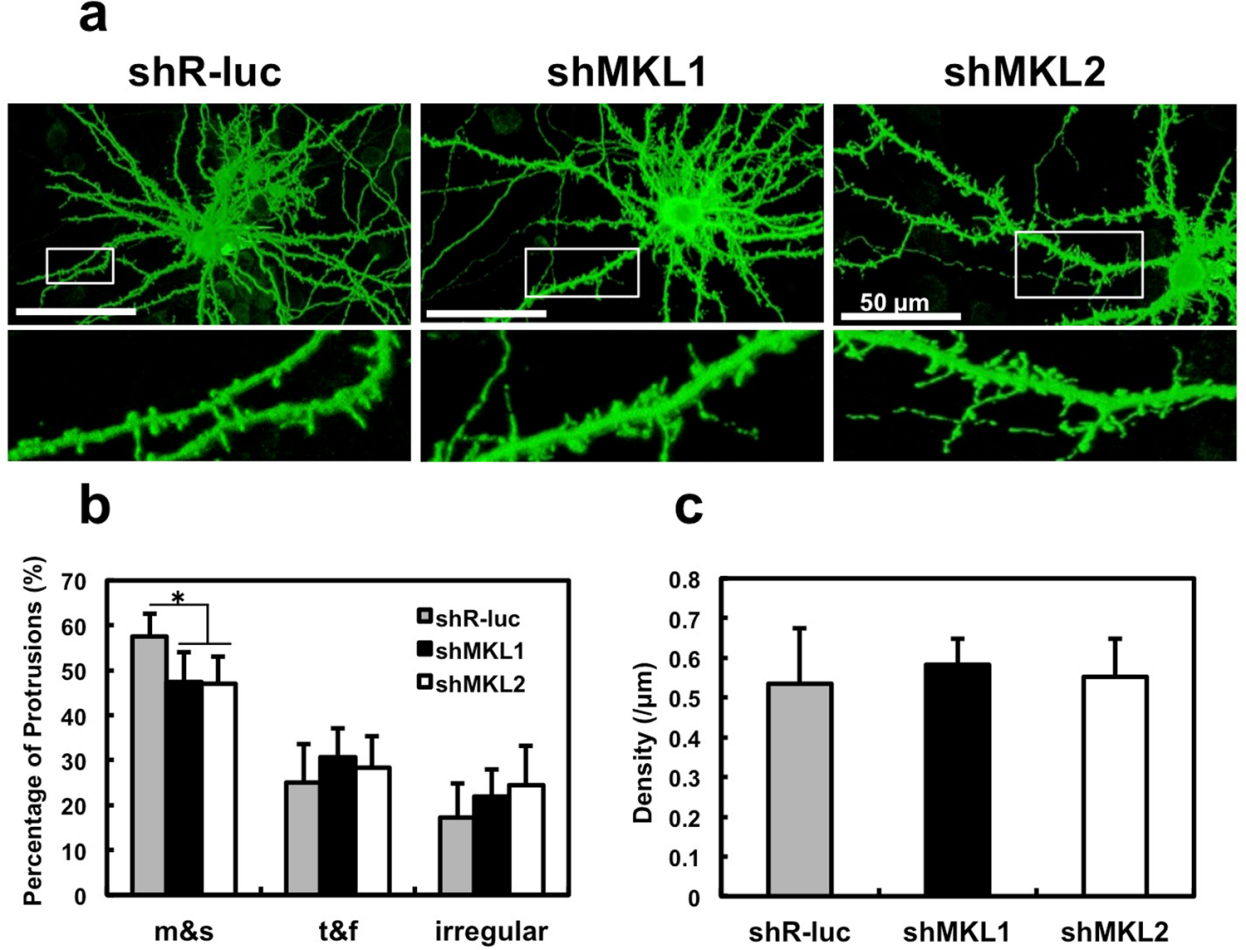
Effect of MKL1 or MKL2 knock-down on dendritic spine morphology and density in cortical neurons. (**a**) Spine morphology of cortical neurons transfected with pEGFP-C1 (1 μg/well) and shR-luc, sh*MKL1*, or sh*MKL2* (1 μg/well). Transfection was performed at 16 days in culture. Neurons (21 days in culture) were immunostained using anti-GFP antibody. (**b**,**c**) Spine morphology (m and s, mushroom or stubby; t and f, thin or filopodia; irregular) and spine density. Graphs show mean ± S.D. from seven independent experiments. The statistical significance of differences (vs. shR-luc) was analysed by ANOVA with the Tukey–Kramer test. *p < 0.05.

**Figure 6:**
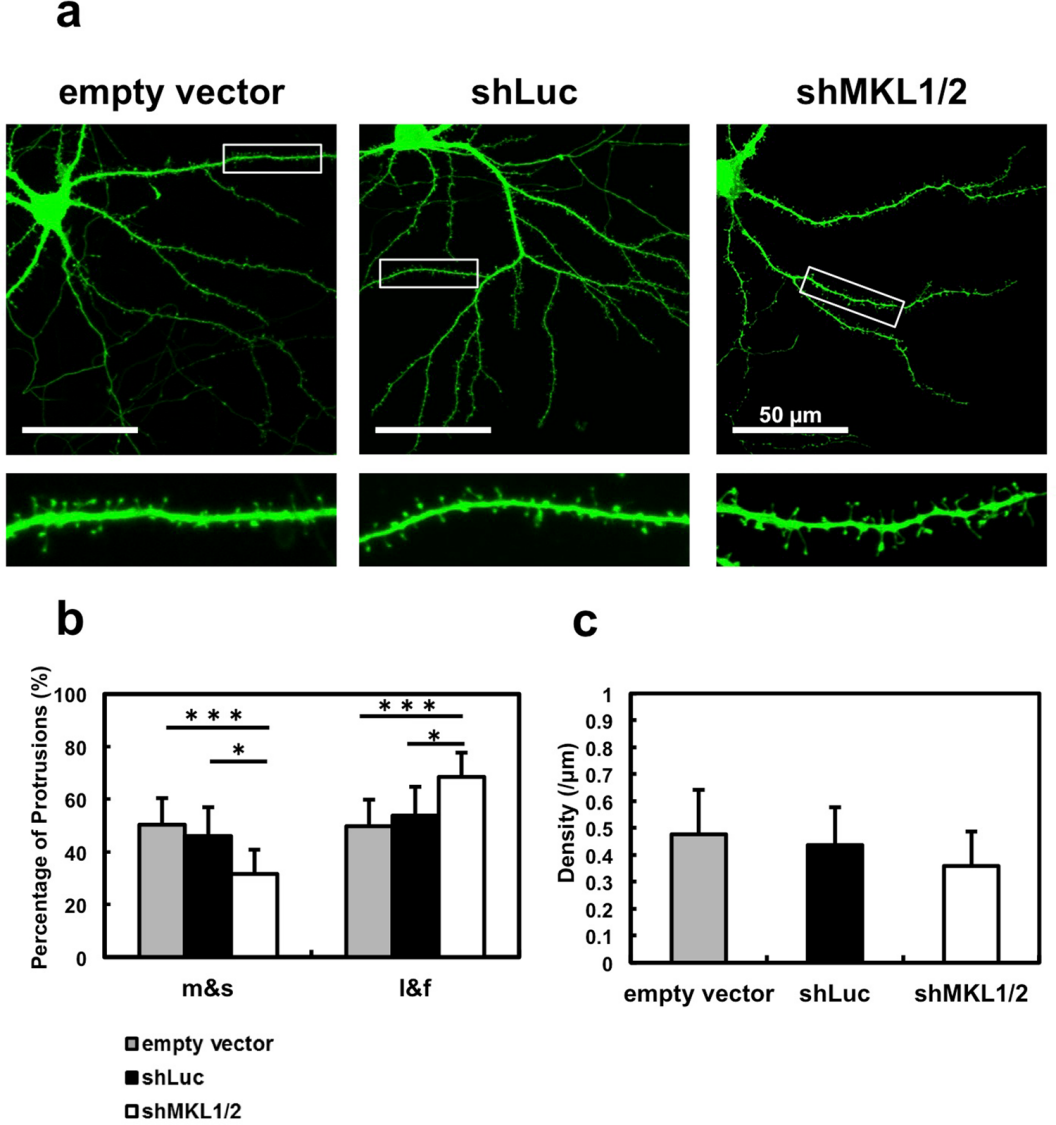
Effect of MKL knock-down on dendritic spine morphology and density in hippocampal neurons. (**a**) Spine morphology of hippocampal neurons transfected with pSynapsinGFP (0.5 μg/well) and 1 μg/well of pSuper (empty vector), shLuc, or sh*MKL1/2*. Transfection was performed at 9 or 10 days *in vitro* (DIV). Neurons (DIV 16) were immunostained using anti-GFP antibody. Dendritic spine morphology was analysed using the semi-automated SpineMagick software. (**b**) The percentage of protrusions clustered into four categories: mushroom, stubby, long, and filopodia (m and s: mushroom or stubby; l and f: long or filopodia). (**c**) Dendritic spine density. Graphs show mean ± S.D. from four independent experiments. The statistical significance of differences was analysed by ANOVA with Dunn’s multiple comparisons test. *p < 0.05, ***p < 0.001.

As shown in Figs 3 and 4, we detected MKL1 and MKL2 protein at synapses. To address MKL1 and MKL2 function in spine morphology, we transfected cultured cortical neurons with GFP and a series of small hairpin (sh) RNA vectors, and analysed spine density and morphology (Fig. 5). Representative images are shown in Fig. 5a. Expression of *MKL1* or *MKL2* shRNA in cortical neurons significantly decreased the percentage of dendritic spines with mushroom- or stubby-shaped structures (Fig. 5b, m and s in left panel). Although there were no significant changes in the percentage of spines with thin- or filopodia-like shapes (Fig. 5b, t and f in left panel) and irregular shapes (Fig. 5b, irregular in left panel), they tended to increase in cells expressing *MKL1* or *MKL2* shRNA. In contrast, *MKL1* and *MKL2* shRNA expression had no effect on dendritic spine density (Fig. 5c, right panel). To confirm the role of MKL in regulation of spine morphology, hippocampal cultures were used (Fig. 6). Expression of sh*MKL1* and sh*MKL2* (whose sequences are different from those used in Fig. 5) significantly decreased the percentage of dendritic spines with mushroom- or stubby-shaped structures, and increased the percentage of long, filopodia spines (Fig. 6b and Supplementary Fig. S4a). Consistent with the results in cortical neurons, sh*MKL1* and sh*MKL2* did not affect spine density (Fig. 6c and Supplementary Fig. S4b). Taken together, these results indicate that MKL1 and MKL2 may play a key role in promoting dendritic spine maturation.

## Discussion

In this study, we initially generated and evaluated MKL1- or MKL2-specific antibodies. Our first biochemical analysis by western blotting showed that MKL1 and MKL2 antibodies do not cross-react with MKL2 and MKL1, respectively, and specifically recognise MKL1 and MKL2 (Fig. 1a). Using cell lysates from rat cortical neurons, we found that these antibodies react with rat MKL1 and MKL2 (Fig. 1b). Antibody absorption tests with antigen-conjugated Sepharose beads and immunocytochemical knock-down experiments with sh*MKL1* and sh*MKL2* revealed that immunostained signals were derived from endogenous mouse and rat MKL1 and MKL2 (Supplementary Figs S1and S2).

Our previous biochemical analyses with commercially available antibodies indicated that MKL is localised in both the cytoplasm and nucleus of cortical neurons^18^. Consistent with this, we confirmed nucleocytoplasmic staining of NIH3T3 cells and cortical neurons with our MKL1 and MKL2 antibodies (Figs 2 and 4a). Furthermore, our antibodies stain nuclear MKL1 and MKL2 due to excessive activation of Rho signalling by constitutively active mDia in NIH3T3 cells (Fig. 2a). The quantitative data suggest that MKL1 and MKL2 efficiently translocate into the nucleus in NIH3T3 cells expressing camDia (Supplementary Fig. S3). In contrast, in cortical neurons, the levels of MKL1 and MKL2, which were located in the nucleus only, were not altered much by camDia (Supplementary Fig. S3). Rather, the levels in both the nucleus and cytoplasm were up-regulated, and the levels in the cytoplasm were down-regulated (Fig. 2c and Supplementary Fig. S3). Previously, we detected nuclear MKL in cells expressing camDia using another antibody that recognised the N-terminal of MKL^27^. Although our antibodies distinctly recognise the C-termini of MKL1 and MKL2, we also successfully detected alteration of cellular localisation of MKL induced by camDia. MKL in NIH3T3 cells tends to more readily translocate than MKL in cortical neurons. Efficient nuclear translocation of MKL1 and MKL2 might be dependent upon cell type. Taken together, these results indicate that our antibodies are suitable for studying cellular localisation and expression of MKL1 and MKL2.

Next, we performed western blotting by subcellular fractionated proteins (Fig. 3) and immunostaining (Fig. 4). Western blotting shows faint MKL1 and MKL2 bands in cytoplasmic fractions compared with those in CSV and PSD fractions. Contradictorily, cytoplasmic staining was predominant, rather than synaptic staining (Fig. 4). There is one possibility to explain this inconsistency. The CSV and PSD fractions include not only CSV and PSD but also a variety of membrane components. With immunostaining, there are a lot of puncta in cell bodies of cortical neurons. Collectively, punctate cell body signals may imply that MKL1 and MKL2 translocate from the cytoplasm to synapses or dendrites or from synapses or dendrites to the cytoplasm via vesicular (membrane) transport. Thus, membrane vesicles containing MKL1 and MKL2 in cell bodies might have been concentrated and detected in CSV and PSD fractions in this study. So far, we do not know what the punctate signals in cell bodies and dendrites are. It would be interesting to clarify the identity of the punctate signal, and further studies are required to address this issue.

We immunocytochemically confirmed that some MKL1 and MKL2 puncta are localised postsynaptically although not all MKL1 and MKL2 puncta are colocalised with PSD-95- positive puncta (Fig. 4). Considering our biochemical analysis with subcellular fractions (Fig. 3), postsynaptic localisation of MKL1 and MKL2 is, at least in part, observed. These findings suggest that MKL1 and MKL2 play important roles in synaptic transmission and function at the PSD. To investigate this possibility, we determined if MKL1 and MKL2 are involved in modification of dendritic spine morphology (Fig. 5). Sh*MKL1* and sh*MKL2* reduced the percentage of mushroom- or stubby-shaped structures, suggesting that MKL may be involved in spine maturation. Consistent with the results obtained in cortical neurons, hippocampal neurons expressing sh*MKL1* or -*2* reduced the percentage of mushroom- and stubby-shaped spines (m and s) and did not affect dendritic spine density (Fig. 6b and c). Furthermore, sh*MKL1* or -*2* expression increased long- and filopodia-shaped spines (l and f), supporting our finding that MKL1 and MKL2 influence spine maturation (Fig. 6b). To exclude the possibility of shRNA off-target effects, we used a separate set of sh*MKL*1 and sh*MKL*2 for morphological analyses. As shown in Figs 5,6 and Supplementary Figure S4, we obtained the same results with shRNAs targeting diverse sequences, providing convincing evidence that our results are due to MKL1- or MKL2-specific knock-down and not off-target effects. Taken together, MKL1 and MKL2 may act at the PSD to promote dendritic spine maturation. Since MKL1 and MKL2 contain actin-binding motifs, they can bind to G-actin^16^. In contrast, MKL1 can bind to filamin A as an F-actin binding protein^28^. Moreover, F-actin polymerisation is crucial for dendritic spine maturation^29^. Therefore, MKL1 and MKL2 may act as regulators for actin-based alteration of dendritic spine morphology at the PSD. Since MKL1 and MKL2 are SRF transcriptional cofactors, it is possible that MKL1 and MKL2 promote dendritic spine maturation at the transcriptional level. Indeed, microarray analysis of conditional *MKL1* and *MKL2* knock-out in mouse brain demonstrates regulation of a subset of genes^20^. Some of them (including actin, gelsolin, and pctaire1) are related to cytoskeletal regulation^20^. So far, we cannot exclude the possibility that MKL1 and MKL2 promote spine maturation via cytoskeletal gene regulation.

As well as the presence of MKL in the PSD fraction, MKL was detected in CSV and SM fractions (Fig. 3). To clarify this significance, future studies may be required.

Aberrant dendritic spine morphology is associated with neurological disorders including schizophrenia and ASD^30^. Corroboratively, SNPs of the *mkl1* gene are found in patients with schizophrenia^21,22^. In addition, *mkl2* gene mutations or SNPs are candidates for ASD^23,24^. Therefore, these mutations may disturb MKL1 and MKL2 function at synapses.

In this study, using our newly-generated antibodies, we show that MKL1 and MKL2 are localised at synapses and may contribute to spine maturation. Our results provide improved understanding of the regulatory mechanism mediated by MKL family members in brain function and disorders.

## Methods

### Animals

Sprague-Dawley dams for primary cultured cortical neurons were purchased from Japan SLC Inc. (Hamamatsu, Japan). Wistar P1 pups were used for primary cultured hippocampal neurons. All experiments were approved by the Animal Experimentation and Ethics Committee of Kitasato University School of Medicine. Experiments were performed in accordance with the guidelines of the Animal Care and Experimentation Committee of the University of Toyama, Sugitani Campus and Animal Care Welfare Committees at the University of Yamanashi, and according to guidelines of the First Warsaw Ethical Committee on animal research, with appropriate permission. Every effort was made to minimise the suffering of animals.

### Plasmids and antibodies

The expression vectors, pFLAG-CMV2 vector (FLAG-empty) and pCMV-HA (HA-empty), were purchased from Sigma-Aldrich (St. Louis, MO, USA) and Clontech (Polo Alto, CA, USA), respectively. pFLAG-CMV2-MKL1 vector (FLAG-MKL1) and pCMV-HA-MKL2 vector (HA-MKL2) were previously constructed^18^. The expression vector for enhanced green fluorescent protein (pEGFP-C1) was purchased from Clontech. Vectors for expressing sh*MKL1* and sh*MKL2* were described previously^18^. The control vector for RNA interference experiments, shR-Luc, was constructed by mutagenesis of pSUPER using the KOD-Plus Mutagenesis Kit (Toyobo, Osaka, Japan) with appropriate primers: 5′-GAGAGTAGGAGTAGTGAAAGGCCTTTTTAAGCTTATCGATACCGTCGACCTC-3′ and 5′-TTGAAGTAGGAGTAGTGAAAGGCCGGGGATCTGTGGTCTCATACAGAACT-3′.

For observation of nuclear translocation of endogenous MKL1 and MKL2, GFP-tagged constitutively active mDia constructs were used, which were a gift from Shuh Narumiya. For spine density and morphological analyses, in addition to vectors expressing sh*MKL1* and sh*MKL2*^18^, pSynapsinGFP with pSUPER (empty vector), pTRIP-shLuc (shLuc; where shLuc is a shRNA targeting luciferase), or pLKO-shMKL1/2 (sh*MKL1*/*2*), which has a different sequence, were used. pLKO.1-MKL1/2 shRNA was a gift from Ron Prywes (Addgene plasmid # 27161)^31^. pLKO.1-MKL1/2 shRNA shows 100% similarity to rat MKL2 and 95% similarity to rat MKL1. The vector expressing sh*MKL1*, which targets 5′-CCAAGGAGCTGAAGCCAAA-3′, was previously described^19^ It was used for analysing hippocampal neuronal morphology (Supplementary Fig. S4).

The following antibodies were used at the indicated dilutions: goat CF594-conjugated anti-guinea pig IgG (1:1000, 20118–1; Biotium, Hayward, CA, USA), goat CF488A-conjugated anti-rabbit IgG (1:1000, 20012–1; Biotium), goat CF488A-conjugated anti-mouse IgG (1:1000, 20010–1; Biotium;), and Alexa Fluor 488 (1:200, A-10680; Invitrogen, Carlsbad, CA, USA). Rabbit polyclonal anti-GFP antibody was purchased from Medical & Biological Laboratories (1:1000, 598; Nagoya, Japan). Donkey horseradish peroxidase (HRP)-conjugated anti-rabbit IgG and sheep HRP-conjugated anti-mouse IgG were purchased from GE Healthcare (1:5000, NA934VS and NA931VS; Little Chalfont, UK). Rabbit HRP-conjugated anti-guinea pig IgG was from Invitrogen (1:5000, 614620). Anti-MKL1 and anti-MKL2 produced in guinea pigs (see “Generation of antibodies”) were used for immunostaining (MKL1, 1.14 μg/ml for cortical neurons and 0.114 μg/ml for NIH3T3 cells; MKL2, 2.5 μg/ml for cortical neurons and 0.5 μg/ml for NIH3T3 cells) and western blotting (MKL1, 0.114 μg/ml; MKL2, 0.5 μg/ml). The following mouse monoclonal antibodies were used: anti-FLAG (1:1000, F3165; Sigma-Aldrich), anti-HA (1:1000, H9658; Sigma-Aldrich), anti-PSD-95 (1:1000 for western blotting, ab12093; Abcam, Cambridge, MA, USA), anti-PSD-95 (1:500 for immunostaining, ADI-VAM-PS002; ENZO Life Sciences, Exeter, UK), anti-α-tubulin (1:1000, T9026; Sigma-Aldrich), anti-synaptophysin antibody (1:1000 for western blotting and 1:200 for immunostaining, s5768; Sigma-Aldrich), and anti-GFP antibody (in Fig. 6 and Supplementary Fig. S4) (1:500, MAB3580; Merck, Kenilworth, NJ, USA).

### Western blotting

Western blotting was performed as previously described^18^. Protein detection was performed using the enhanced chemiluminescence protocol (ECL Western blotting detection reagents, GE Healthcare; ImmunoStar LD, ImmunoStar Zeta, Wako Pure Chemical Industries, Ltd., Osaka, Japan).

### Generation of antibodies

Generation of antibodies against MKL1 or MKL2 was as follows. The C-terminal regions of mouse MKL1 (333 amino acid residues) and mouse MKL2 (333 amino acid residues) proteins were bacterially expressed as maltose-binding protein (MBP) fusion proteins and purified using amylose-resin (New England Biolabs, Ipswich, MA, USA). MBP-MKL1 or MBP-MKL2 fusion proteins were conjugated with Freund’s adjuvant and subcutaneously injected into guinea pigs five times at 2-week intervals. After sera were incubated with MBP to eliminate immunoglobulins against MBP, specific antibodies were further affinity-purified using MBP-MKL1 or MBP-MKL2 fusion proteins coupled to CNBr-activated Sepharose 4B (GE Healthcare).

### Cell culture

The culture conditions and reagents for NIH3T3 cells were previously described^27^_._.

Culture conditions and reagents for dissociating cortical cells were previously described^32,33^. Half of the medium was replaced every 3 days. To observe synaptic localisation of MKL and dendritic spine morphology, the B27-Electrophysiology Kit containing 0.5 mM GlutaMAX-I supplement and 2 µg/mL gentamicin (Gibco/Life Technologies, Carlsbad, CA, USA) was used as the culture medium. Cells were plated at a density of 3 × 10^5^ cells/well onto 18-mm circle coverslips coated with poly-D-lysine, and placed in 12-well plates. Half of the medium was replaced at 1, 4, 7, 10, 13, 16, and 17 days (Fig. 4a and b), 1, 4, 7, 10, 13, 16, and 19 days (Fig. 4c and d) and 1, 4, 7, 10, 13, 15, and 18 days (Fig. 5). Hippocampal cultures were prepared from rat pups (P1), and cultured in neurobasal medium (Life Technologies) supplemented with B27 (Life Technologies), 0.5 mM glutamine, 2 mM glutamate, and 50 μg/ml penicillin-streptomycin.

### Transfection in NIH3T3 cells, and rat cortical and hippocampal neurons

Transfection of NIH3T3 cells was performed using Lipofectamine (Invitrogen) and Plus reagents (Invitrogen), as previously described^18,27^. Cortical neurons cultured for 7 (Fig. 2), 16 (Fig. 5) or 18 (Fig. 4) days were transfected by the calcium phosphate precipitation method with minor modifications^18^. Hippocampal neurons were transfected with plasmid DNA at 9–10 days *in vitro* using Lipofectamine® 2000 (Life Technologies).

### Immunostaining

Immunostaining methods of NIH3T3 cells and cortical cultures were performed as previously described^27,32,33^.

### Purification of synaptic proteins

Subcellular fractionation of 6-week-old male rat brain was performed as previously described^34^.

### Morphological analysis

To observe dendritic spine morphology of cortical neurons, an average of 40 neurons were selected and images containing two full-length dendrites collected. The number of spines was quantified and classified into three groups: mushroom/stubby (width of spine head is longer than spine length), thin/filopodia (spine length is longer than width of spine head), and irregular (branched spine or protrusion from spine head or much larger spine size). Simultaneously, spine density was counted. Images were obtained by microscopy (LSM700; Carl-Zeiss, Oberkochen, Germany).

To observe dendritic spine morphology of hippocampal neurons, fluorescent images were captured with a Leica SP8 SMD confocal microscope (Wetzlar, Germany). For spine density and morphological analyses, only transfected neurons that were distinct from other transfected cells were evaluated with SpineMagick Software^35^. Spine clustering was performed using custom scripts^36^.

### Statistical analysis

Statistical significance of treatment effects was analysed by analysis of variance (ANOVA) with Tukey–Kramer and Dunn’s multiple comparison tests using Prism software (GraphPad, La Jolla, CA, USA). Detailed analyses are outlined in figure legends.

## Electronic supplementary material

Supplementary Information
