# Supplementary material for: Synaptic localisation of SRF coactivators, MKL1 and MKL2, and their role in dendritic spine morphology
Source: Sci Rep. 2018 Jan 15;8:727. doi: 10.1038/s41598-017-18905-7 (PMC5768758; doi:10.1038/s41598-017-18905-7)
Supplement: Supplementary file 1 — Supplementary Information [file 41598_2017_18905_MOESM1_ESM.pdf]

Supplementary Information:

Title: Synaptic localisation of SRF coactivators, MKL1 and MKL2, and their role in dendritic spine morphology

Authors: Marisa Kaneda[1], Hiroyuki Sakagami[2], Yamato Hida[3], Toshihisa Ohtsuka[3], Natsumi Satou[1], Yuta Ishibashi[1], Mamoru Fukuchi[1,4], Anna Krysiak[5], Mitsuru Ishikawa[1,6], Daisuke Ihara[1], Katarzyna Kalita[5,*], and Akiko Tabuchi[1,*]

Affiliations:

[1]Laboratory of Molecular Neurobiology, Graduate School of Medicine and Pharmaceutical Sciences, University of Toyama, 2630 Sugitani, Toyama 930-0194, Japan

[2]Department of Anatomy, Kitasato University School of Medicine, Sagamihara, Kanagawa 252-0734, Japan

[3]Department of Biochemistry, Graduate School of Medicine, University of Yamanashi, 1110 Shimokato, Chuo, Yamanashi 409-3898, Japan

[4]Current affiliation: Laboratory of Molecular Neuroscience, Faculty of Pharmacy, Takasaki University of Health and Welfare, 60 Nakaorui-machi, Takasaki, Gunma 370-0033, Japan

[5]Laboratory of Neurobiology, Department of Molecular and Cellular Neurobiology, Nencki Institute of Experimental Biology, Polish Academy of Sciences, 3 Pasteur Street, 02-093 Warsaw, Poland

[6]Current affiliation: Department of Physiology, Keio University, School of Medicine, 35 Shinanomachi, Shinjuku-ku, Tokyo 160-8582, Japan

*To whom correspondence should be addressed:

Katarzyna Kalita, Ph.D., Laboratory of Neurobiology, Department of Molecular and Cellular Neurobiology, Nencki Institute of Experimental Biology, Polish Academy of Sciences, 3 Pasteur Street, 02-093 Warsaw, Poland

Tel.: +48-22-5892-339 Fax: +48-22-822-53-42, Email: k.kalita@nencki.gov.pl

and

Akiko Tabuchi, Ph.D., Laboratory of Molecular Neurobiology, Graduate School of Medicine and Pharmaceutical Sciences, University of Toyama, 2630 Sugitani, Toyama 930-0194, Japan

Tel.: +81-76-434-7536, Fax: +81-76-434-5048, Email: atabuchi@pha.u-toyama.ac.jp

Methods

Animals

Information is included in the main text.

Plasmids and antibodies

Information on other vectors and antibodies is included in the main text.

Cell culture

The culture conditions and reagents for NIH3T3 cells and cortical neurons were previously described[1, 2]. For knock-down experiments in cortical neurons, the medium was from the B27-Electrophysiology Kit, and contained 0.5 mM GlutaMAX-I supplement and 2 µg/mL gentamicin (Gibco/Life Technologies, Carlsbad, CA, USA). Half of the medium was replaced at 1, 4, 7, 10, 12, and 15 days.

Transfection into NIH3T3 cells and rat cortical and hippocampal neurons

Information on transfection of NIH3T3 cells is included in the main text. For confirmation of MKL1 and MKL2 antibody specificity, 13-day cultured cortical neurons were transfected using the calcium phosphate precipitation method, as previously described[1].

Information on transfection in hippocampal neurons is included in the main text.

Immunostaining

Immunostaining methods of NIH3T3 cells and cortical cultures were performed as previously described[3].

Morphological analysis

Information is included in the main text.

Western blotting

Information is included in the main text.

Statistical analysis

Statistical significance of treatment effects was analysed by nonparametric Mann Whitney *U* test using Prism software (GraphPad, La Jolla, CA, USA).

Results

Evaluation of new antibodies by immunocytochemistry

We determined if MKL1 and MKL2 antibodies are suitable for immunocytochemistry. To confirm the fluorescent signal arising from endogenous MKL1 and MKL2, and exclude non-specific binding, we performed antibody-absorption tests. Antibodies were treated with Sepharose beads conjugated with MKL1- or MKL2-antigen and the mixture centrifuged. The resulting supernatant was used for immunostaining of NIH3T3 cells or cortical neurons. Fluorescent MKL1 signal was detected in the absence of Sepharose beads (Supplementary Fig. S1a, no beads, left and upper panels) or presence of MKL2-Sepharose beads (Supplementary Fig. S1a, MKL2 beads, left and bottom panels). Moreover, treatment of MKL1-Sepharose beads abolished the signal (Supplementary Fig. S1a, MKL1 beads, left and middle panels). Fluorescent MKL2 signal was detected in the absence of Sepharose beads (Supplementary Fig. S1a, no

beads, right and upper panels) or presence of MKL1-Sepharose beads (Supplementary Fig. S1a, MKL1 beads, right and middle panels). While treatment of MKL2-Sepharose beads abolished the signal (Supplementary Fig. S1a, MKL2 beads, right and bottom panels). The same experiments were performed in cortical neurons, with the same results obtained (Supplementary Fig. S1b). Taken together, these results indicate that the immunopositive signal detected in cells is derived from MKL1 and MKL2 antigens.

Further, we tested whether MKL1 and MKL2 antibodies specifically detect endogenous MKL1 and MKL2. Accordingly, small hairpin (sh) RNAs were expressed in NIH3T3 cells (Supplementary Fig. S2a) or cortical neurons (Supplementary Fig. S2b), and we determined if the fluorescent signal in cells expressing sh*MKL1* and sh*MKL2* was reduced by immunostaining with MKL1- and MKL2-antibodies, respectively. We transfected a series of shRNAs with green fluorescent protein (GFP) vector as a reference. After transfection with a control vector, shR-luc (which targets *Renilla* luciferase), GFP-positive cells were stained with MKL1 and MKL2 antibodies (Supplementary Fig. S2a, shR-luc, left and right panels). Transfection of cells with sh*MKL1* vector and GFP resulted in decreased signal after staining with MKL1 antibody (Supplementary Fig. S2a, shMKL1, left panel). In contrast, cells expressing GFP and sh*MKL1* were stained with MKL2 antibody (Supplementary Fig. S2a, shMKL1, right panel). Transfection of cells with sh*MKL2* vector and GFP resulted in decreased signal after staining with MKL2 antibody (Supplementary Fig. S2a, sh*MKL2*, right panel). However, cells expressing GFP and sh*MKL2* were stained with MKL1 antibody (Supplementary Fig. S2a, shMKL2, left panel). The same experiments were performed in cortical neurons, with the same results obtained (Supplementary Fig. S2b).

Therefore, we can confirm that MKL1 and MKL2 antibodies specifically detect endogenous MKL1 and MKL2, respectively, and are suitable for immunostaining.

Quantitative analysis of nuclear translocation of MKL1 and MKL2 in NIH3T3 cells and cortical neurons.

Using the data from Figure 2 in the main text, the cellular localisation of MKL1 and MKL2 in cells expressing constitutively active mDia (camDia) and constitutively inactive mDia (ciamDia) are represented (Supplementary Fig. S3). Details of these results can be found in the main text.

Another shRNA for MKL1 decreased dendritic spine maturation.

We examined the effects of different *MKL1* and *MKL2* shRNAs on dendritic spine morphology (Figs. 5 and 6). To exclude possible off-target effects of *MKL* shRNA, we used a *MKL1* shRNA with a different target sequence. As shown in Supplementary Figure S4a, *shMKL1* expression significantly reduced the percentage of mushroom- or stubby-shaped spines and increased long- or filopodia-shaped spines. Dendritic spine density was not changed by expression of sh*MKL1* (Supplementary Fig. S4b).

References

1. Ishikawa, M. *et al*. Involvement of the serum response factor coactivator megakaryoblastic leukemia (MKL) in the activin-regulated dendritic complexity of rat cortical neurons. J. Biol. Chem. **285**, 32734-32743 (2010).
2. Tabuchi, A. *et al.* Nuclear translocation of the SRF co-activator MAL in cortical neurons: role of RhoA signalling. *J. Neurochem.* **94**, 169-180 (2005).

3. Ishikawa, M. *et al.* Identification, expression and characterization of rat isoforms of the serum response factor (SRF) coactivator MKL1. *FEBS Open Bio.* **3**, 387-393 (2013).

Figure Legends

Figure S1. Immunocytochemical signal of MKL1 and MKL2 is abolished by antibody absorption.

NIH3T3 cells (a) and cortical neurons at 10 days in culture (b) were fixed and subjected to immunostaining using anti-MKL1 and anti-MKL2 primary antibodies. No bead control indicates use of primary antibody, while MKL1 beads and MKL2 beads indicate use of primary antibodies preincubated with MKL1- and MKL2-conjugated Sepharose 4B, respectively.

Figure S2. Knock-down experiment to confirm detection of endogenous MKL1 and MKL2.

The pSUPER-shR-luc vector, pSUPER-shMKL1 vector or pSUPER-shMKL2 vector and pEGFP-C1 vector were co-transfected into NIH3T3 cells (a) by lipofection method and rat cortical neurons (13 days in culture) (b) by calcium phosphate precipitation method (GFP vector 0.5 μg/well, shRNA 1.5 μg/well). Cells were immunostained 96 hours (NIH3T3 cells) or 96 hours (cortical neurons) after transfection.

Figure S3. Quantitative analysis of the cellular distribution of MKL1 and MKL2 in NIH3T3 cells and cortical neurons.

NIH3T3 cells expressing constitutively active mDia (camDia) or constitutively inactive

mDia (ciamDia) were immunostained with GFP and MKL1 (a: NIH3T3 camDia-MKL1 or b: NIH3T3 ciamDia-MKL1) or MKL2 (c: NIH3T3 camDia-MKL2 or d: NIH3T3 ciamDia-MKL2) antibodies. Cortical neurons expressing constitutively active mDia (camDia) or constitutively inactive mDia (ciamDia) were immunostained with GFP and MKL1 (e: Neuron camDia-MKL1 or f: Neuron ciamDia-MKL1) or MKL2 (g: Neuron camDia-MKL2 or h: Neuron ciamDia-MKL2) antibodies. 'Nucleus' indicates that the nuclear staining was more intense of than that of cytoplasmic staining. 'Cytoplasm' indicates that the cytoplasmic staining was more intense than that of nuclear staining. Where both are indicated the intensity of the nuclear and cytoplasmic staining was the same. Data were collected from 100 cells in one experiment.

Figure S4. Effect of another *MKL1* shRNA on dendritic spine morphology and density in hippocampal neurons.

(a) The percentage of protrusions clustered into four categories: mushroom, stubby, long and filopodia (m and s: mushroom or stubby; l and f: long or filopodia). (b) Dendritic spine density. Graphs show mean ± S.D. (n>7) from two experiments. The statistical significance of differences was analysed by Mann Whitney *U* test. *$p < 0.05$.

Figure S5. Full blot images of Figure 1.

Figure S6. Full blot images of Figure 3.

Figure S1

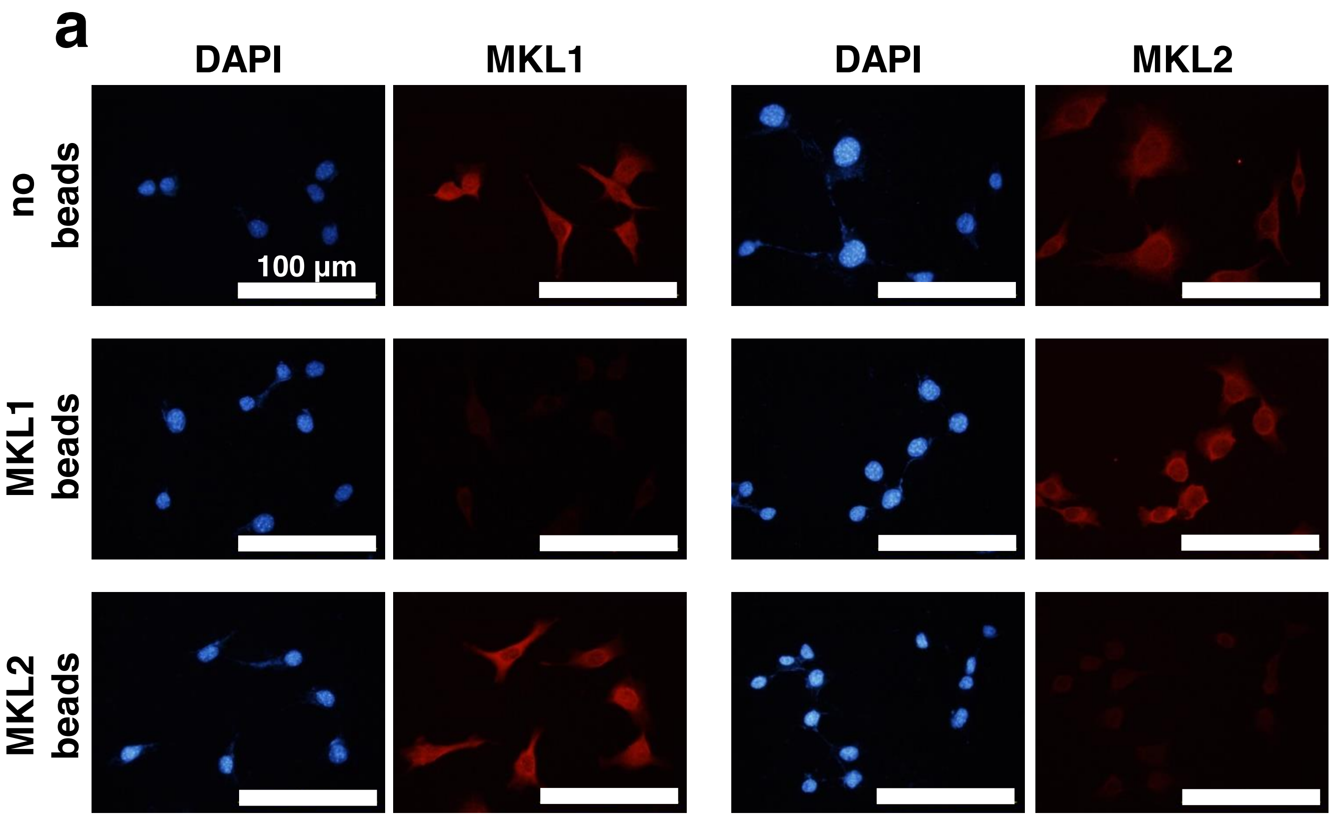
a
DAPI
MKL1
DAPI
MKL2
no beads
MKL1 beads
MKL2 beads
100 µm

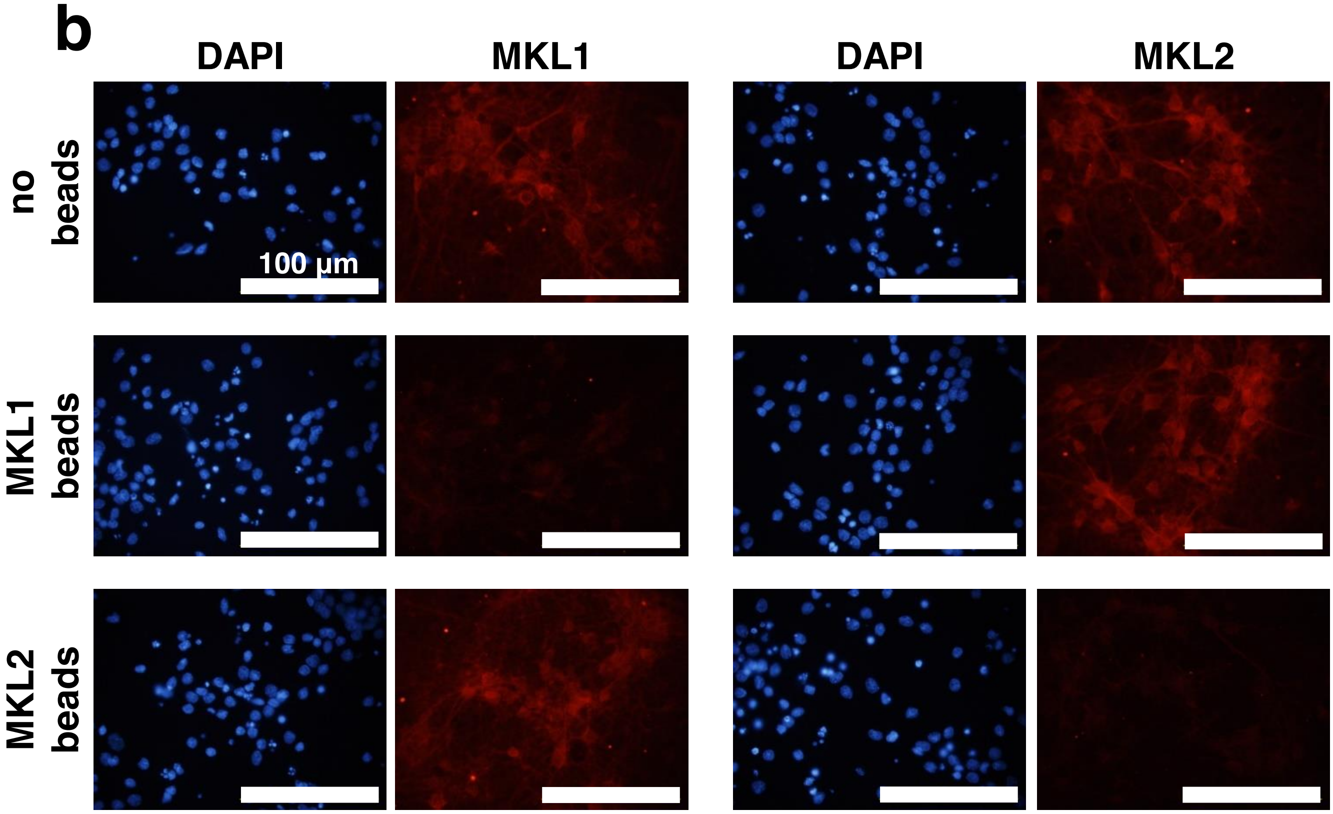
b
DAPI
MKL1
DAPI
MKL2
no beads
MKL1 beads
MKL2 beads
100 µm

Figure S2

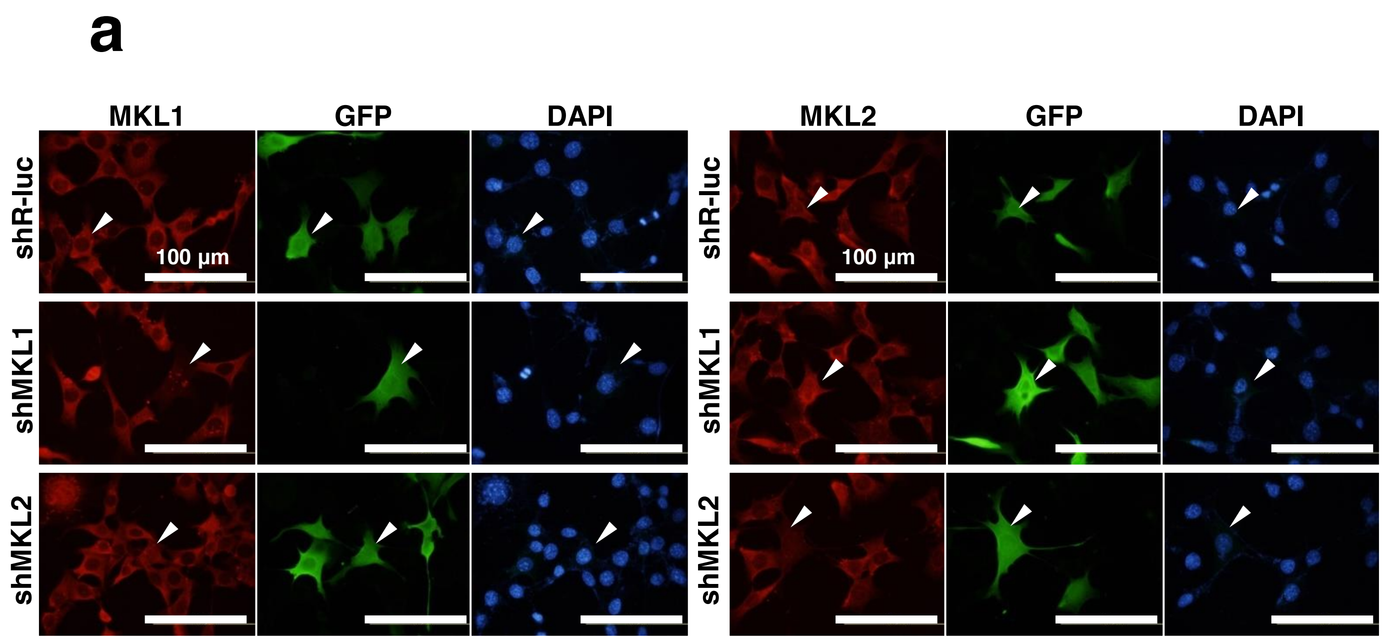
a
MKL1
GFP
DAPI
MKL2
GFP
DAPI
shR-luc
shMKL1
shMKL2
100 µm
100 µm

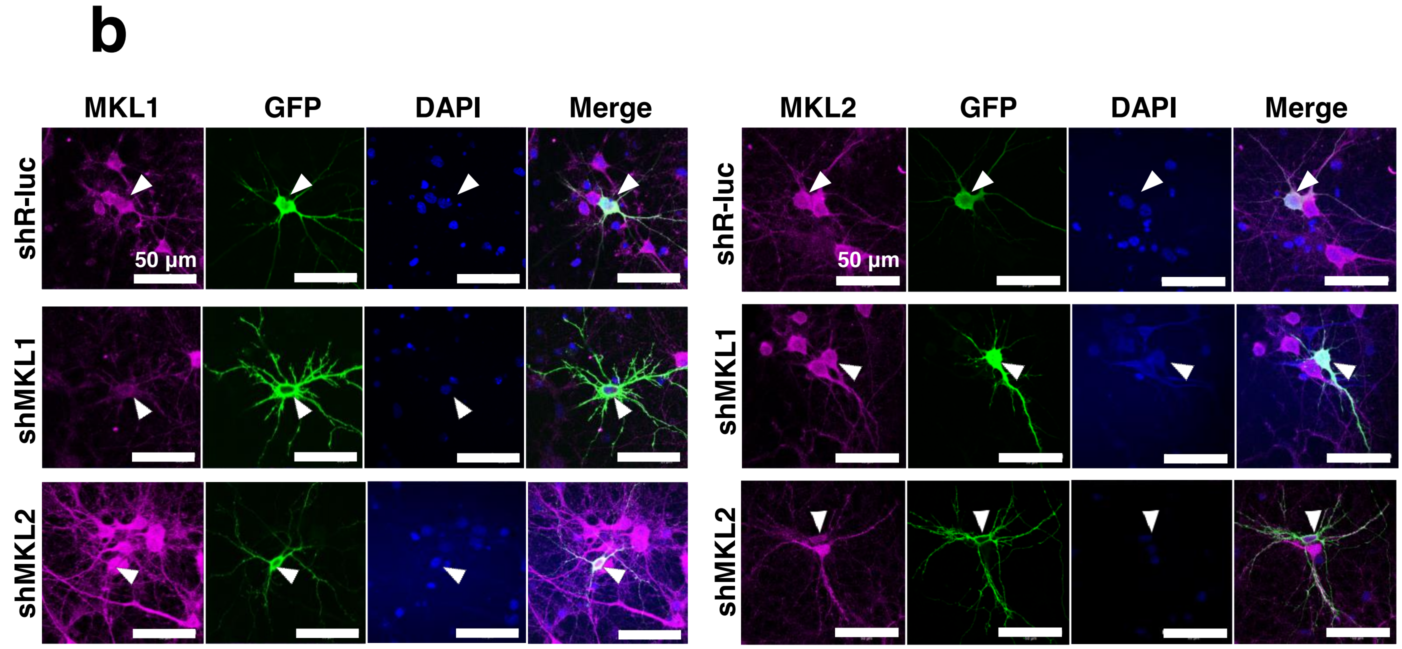
b
MKL1
GFP
DAPI
Merge
MKL2
GFP
DAPI
Merge
shR-luc
shMKL1
shMKL2
50 µm
50 µm

Figure S3

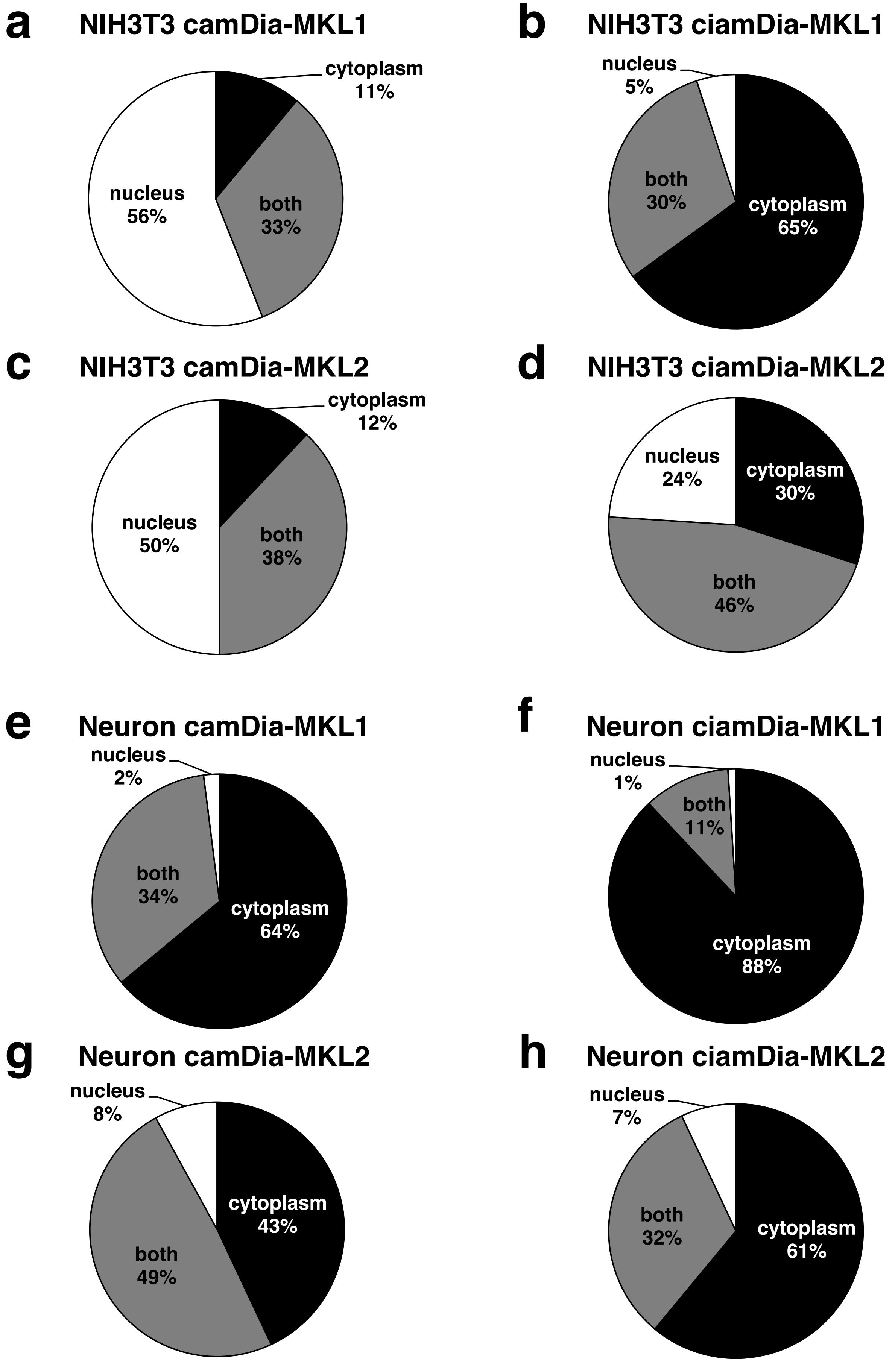
a
NIH3T3 camDia-MKL1
cytoplasm 11%
nucleus 56%
both 33%
b
NIH3T3 ciamDia-MKL1
nucleus 5%
both 30%
cytoplasm 65%
c
NIH3T3 camDia-MKL2
cytoplasm 12%
nucleus 50%
both 38%
d
NIH3T3 ciamDia-MKL2
nucleus 24%
cytoplasm 30%
both 46%
e
Neuron camDia-MKL1
nucleus 2%
both 34%
cytoplasm 64%
f
Neuron ciamDia-MKL1
nucleus 1%
both 11%
cytoplasm 88%
g
Neuron camDia-MKL2
nucleus 8%
cytoplasm 43%
both 49%
h
Neuron ciamDia-MKL2
nucleus 7%
both 32%
cytoplasm 61%

Figure S4

a

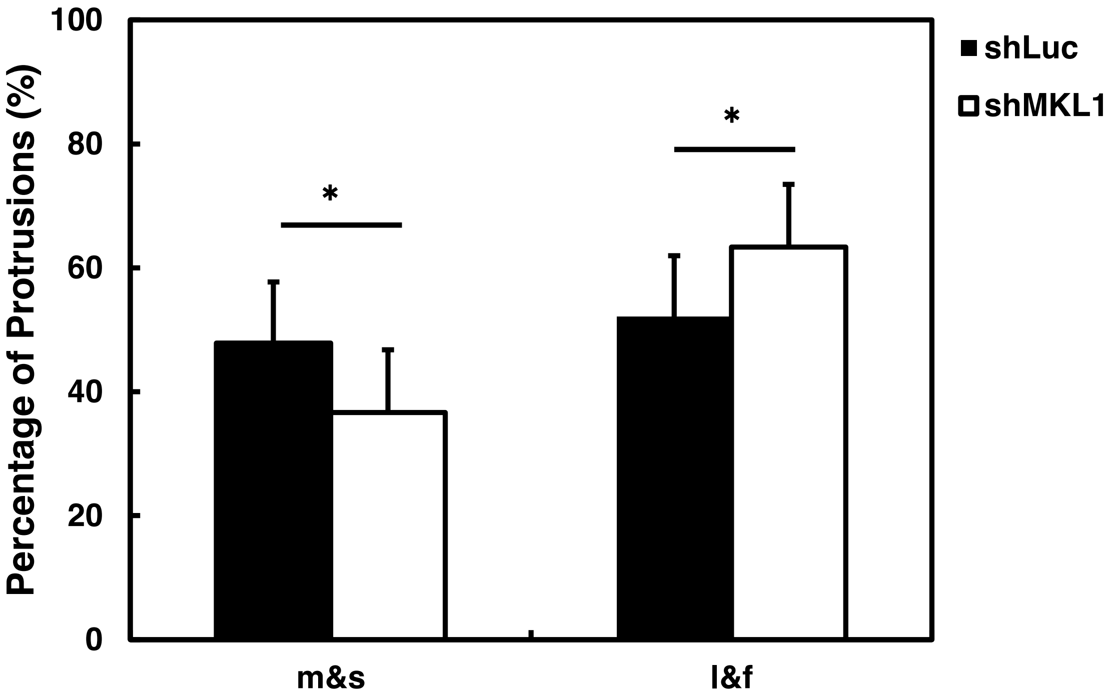

100
80
60
40
20
0
Percentage of Protrusions (%)
*
*
m&s
l&f
shLuc
shMKL1

b

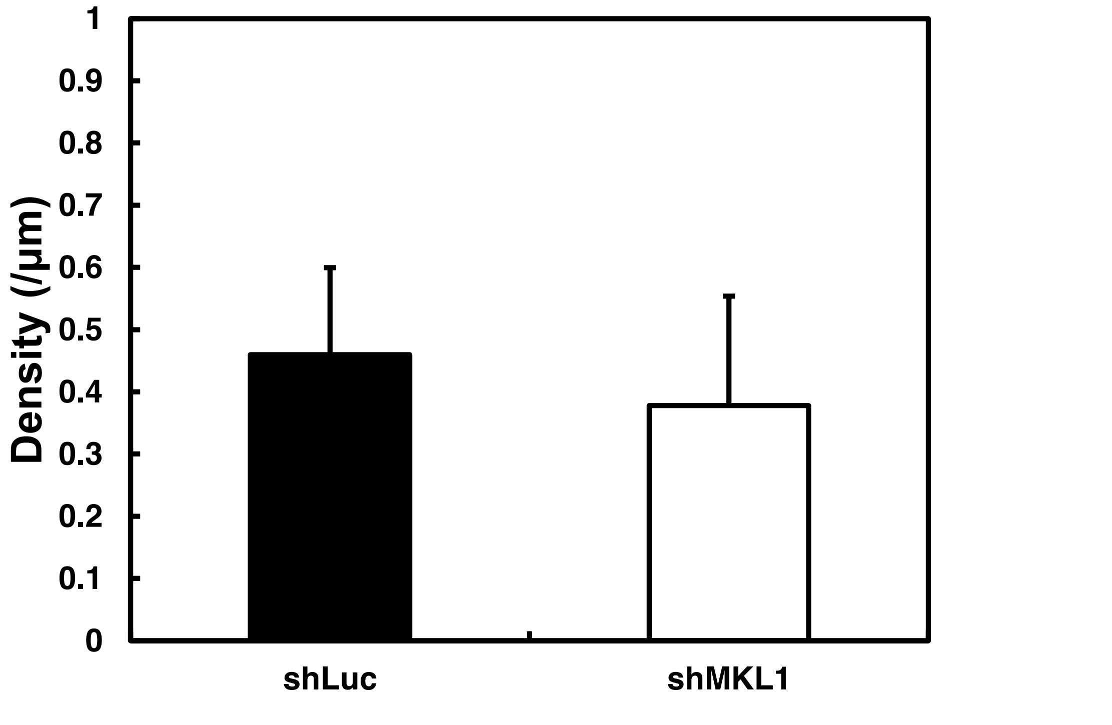

1
0.9
0.8
0.7
0.6
0.5
0.4
0.3
0.2
0.1
0
Density (/µm)
shLuc
shMKL1

Figure S5

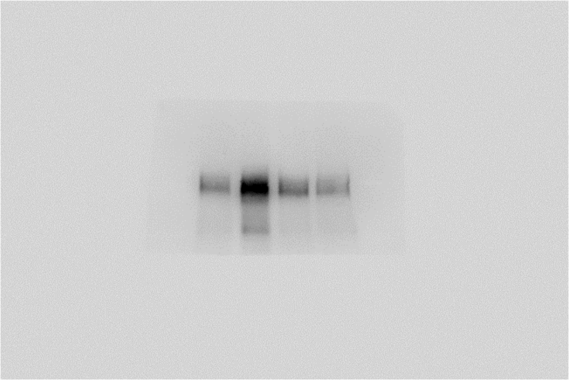

Figure 1a. MKL1

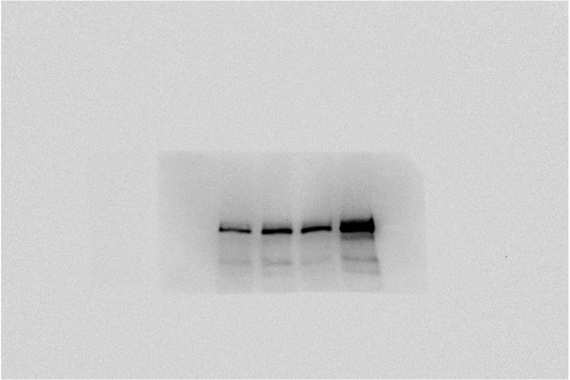

Figure 1a. MKL2

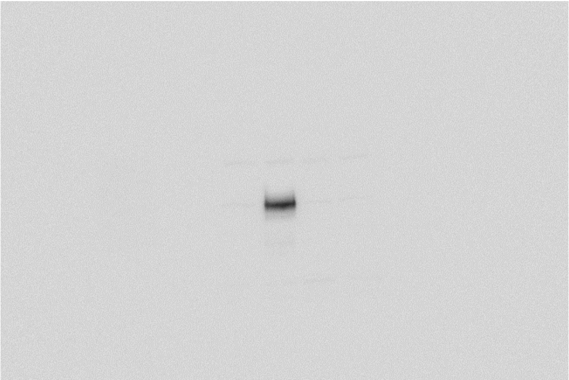

Figure 1a. FLAG

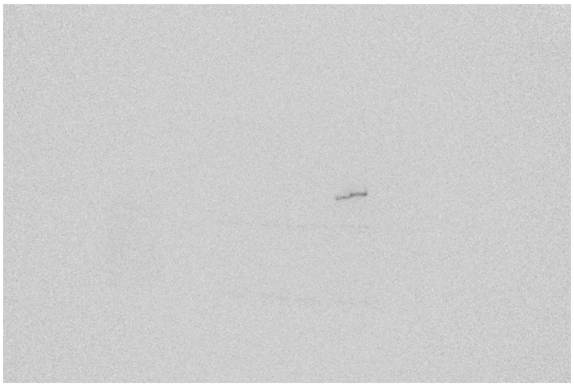

Figure 1a. HA

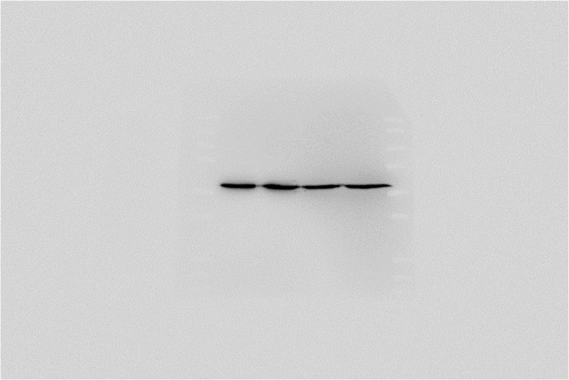

Figure 1a. α-tubulin

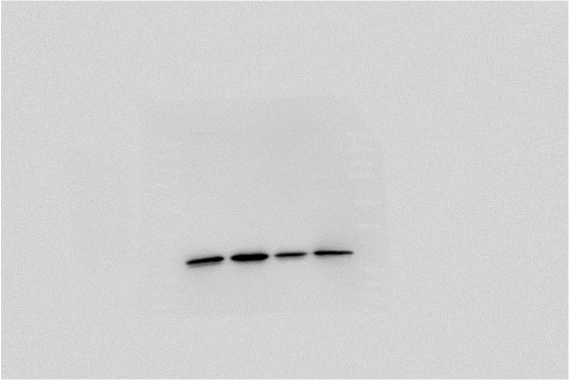

Figure 1a. GFP

Figure S5

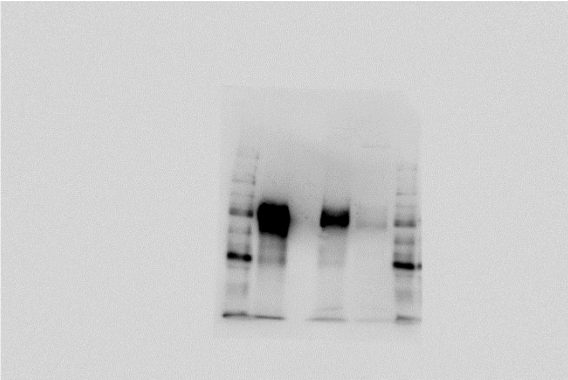

Figure 1b. MKL1

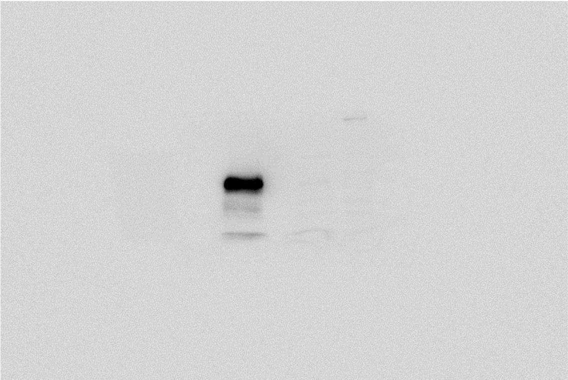

Figure 1b. FLAG

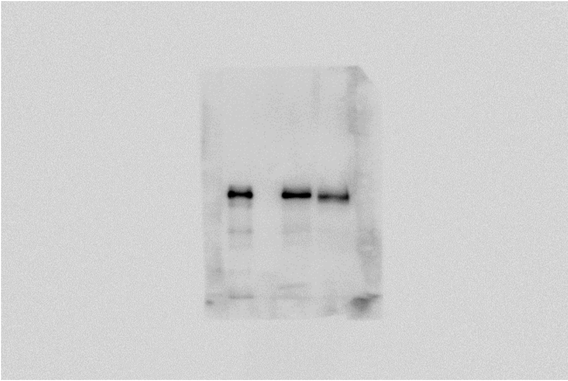

Figure 1b. MKL2

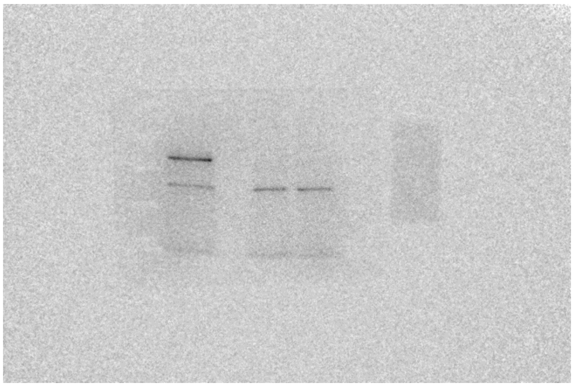

Figure 1b. HA

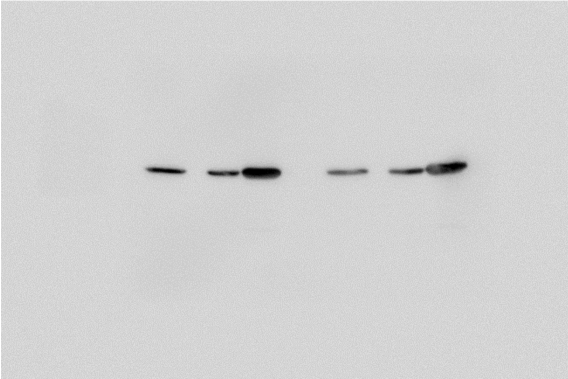

Figure 1b. α-tubulin

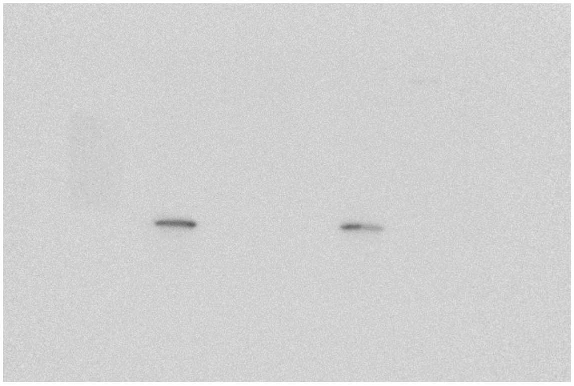

Figure 1b. GFP

Figure S6

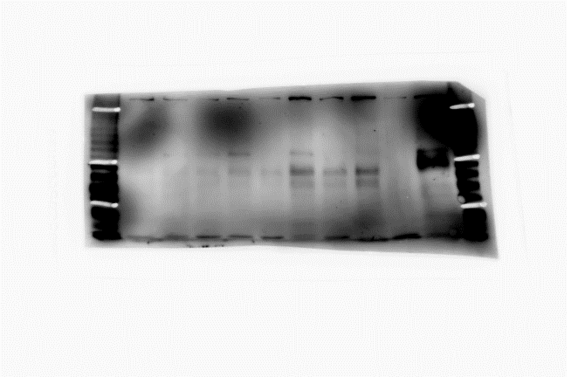

Figure 3. MKL1

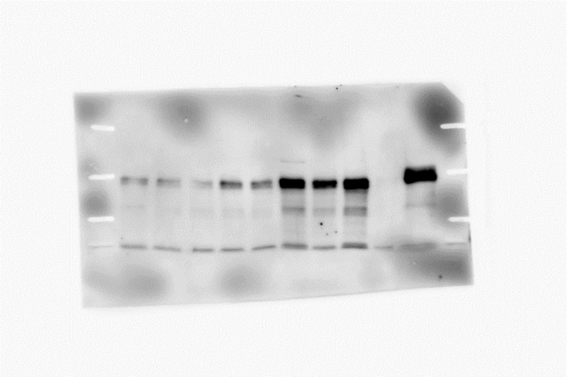

Figure 3. MKL2

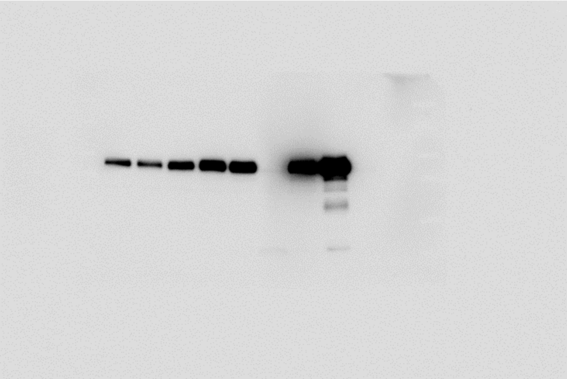

Figure 3. PSD-95

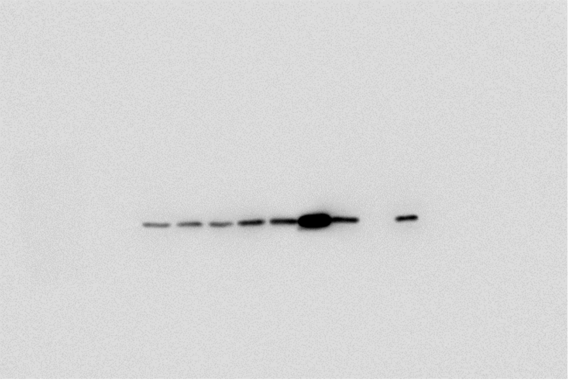

Figure 3. synaptophysin

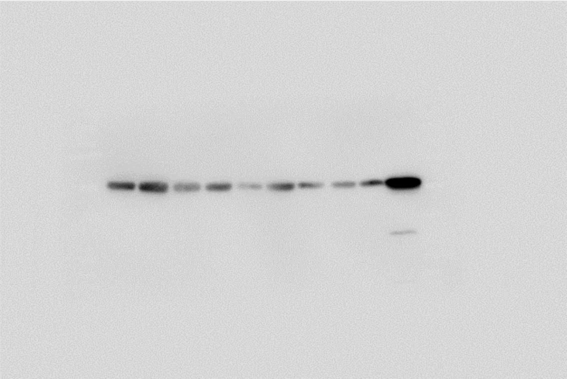

Figure 3. α-tubulin
